# Integrated single-cell and transcriptome sequencing analyses develops a metastasis-based risk score system for prognosis and immunotherapy response in uveal melanoma

**DOI:** 10.3389/fphar.2023.1138452

**Published:** 2023-02-09

**Authors:** Shuting Meng, Tianye Zhu, Zhiwei Fan, Yulan Cheng, Yefeng Dong, Fengxu Wang, Xuehai Wang, Deping Dong, Songtao Yuan, Xinyuan Zhao

**Affiliations:** ^1^ Hai an People’s Hospital, Nantong, China; ^2^ Department of Ophthalmology, The First Affiliated Hospital of Nanjing Medical University, Nanjing, China; ^3^ School of Medicine, Nantong University, Nantong, China; ^4^ Nantong Key Laboratory of Environmental Toxicology, Department of Occupational Medicine and Environmental Toxicology, School of Public Health, Nantong University, Nantong, China

**Keywords:** uveal melanoma, metastasis, prognosis, immunotherapy response, drug sensitivity, areg

## Abstract

**Background:** Uveal melanoma (UM) is the most frequent ocular neoplasm with a strong metastatic ability. The prognostic value of metastasis-associated genes (MAGs) of UM remains unclear. It is urgent to develop a prognostic score system according to the MAGs of UM.

**Methods:** Unsupervised clustering was used to identify MAGs-based molecular subtypes. Cox methods were utilized to generate a prognostic score system. The prognostic ability of the score system was detected by plotting ROC and survival curves. The immune activity and underlying function were depicted by CIBERSORT GSEA algorithms.

**Results:** Gene cluster analysis determined two MAGs-based subclusters in UM, which were remarkably different in clinical outcomes. A risk score system containing six MAGs (COL11A1, AREG, TIMP3, ADAM12, PRRX1 and GAS1) was set up. We employed ssGSEA to compare immune activity and immunocyte infiltration between the two risk groups. Notch, JAK/STAT and mTOR pathways were greatly enriched in the high-risk group. Furthermore, we observed that knockdown of AREG could inhibit UM proliferation and metastasis by *in vitro* assays.

**Conclusion:** The MAGs-based subtype and score system in UM can enhance prognosis assessment, and the core system provides valuable reference for clinical decision-making.

## Introduction

Uveal melanoma (UM), the major primary intraocular malignancy in adults, accounts for 83% of intraocular melanomas. Among them, choroidal, ciliary and iris melanomas account for 85%–90%, 5%–8%, and 3%–5%, respectively (Singh et al., 2011). Patients mostly complain of decreased visual acuity, visual distortion, and loss of visual field, and 30% of patients may not have any ocular symptom, making them highly susceptible to underdiagnosis and misdiagnosis. This highly malignant disease is prone to invasive metastasis, mainly in the liver (89%) (Diener-West et al., 2005). Once metastasized, the prognosis is extremely poor. Currently, UM is mainly treated by ophthalmopexy, local tumor resection, local radiation therapy (external scleral dressing or stereotactic radiation therapy, proton beam treatment) and laser photocoagulation treatment (transpupillary thermal or photodynamic therapy) (Carvajal et al., 2017). The high proliferative activity of UM cells and the extreme susceptibility to extraocular metastasis are the main reasons for the therapeutic difficulty and high mortality of this tumor. However, the current treatments for UM are ineffective against tumor metastasis (Augsburger et al., 2009), so most studies have diverted to immunotherapy. Identification of probable biomarkers of UM may offer key data for early recurrence monitoring or treatment (Bol et al., 2016). Currently, though some key genes and pathways in UM are identified, the prognosis remains unsatisfactory (Xue et al., 2019). Hence, new markers are urgently needed to evaluate the prognosis of UM.

With the fast advancement of immunotherapy recently, the tumor microenvironment (TME) is reportedly pivotal in cancer growth and therapeutic response (Arneth, 2019). Prognostic or predictive biomarkers related to TME may largely help assess tumor prognosis and advance oncology therapies.

TME is a complicated and integrated system consisting of various stromal cells, such as fibroblasts, smooth muscle cells, immune and inflammatory cells, glial cells, adipocytes, and some vascular cells (Song et al., 2021a; Liu et al., 2022a). These cells can be initiated by tumor cells to produce abundant growth factors, cytokines, and stromal degrading enzymes around them, which facilitate the division and invasion of tumor cells (Song et al., 2021b). TME is the material basis for the survival and development of tumor cells, and TME and tumor cells are an interdependent and mutually promoting whole (Chen et al., 2021; Liu et al., 2022b). TME is physiologically characteristic of low oxygen, low pH and high interstitial hydraulic pressure, which provide the necessary material basis for tumor formation, development, invasion, metastasis, drug therapy resistance, and immune response (Watnick, 2012).

Tumor metastasis is a major factor contributing to the poor prognostic outcome of various cancers. There are several theoretical models about the mechanism of tumor metastasis, and the most prevalent one is the epithelial-mesenchymal transition (EMT) theory (Mittal, 2018). This theory suggests that first some cells during tumor metastasis undergo EMT, which causes tumor cells to lose their cell-to-cell adhesion and fall off from the tumor tissues into the blood circulation system. Then the cells flow with the blood to other suitable places for growth (Song et al., 2021c). EMT leads to tumor cytoskeleton rearrangement, reduced cellular rigidity and cell/cellular connectivity, facilitating tumor metastasis and invasion (Davis et al., 2014).

Diverse developmental signaling pathways, such as tumor growth factor (TGF)-β, WNT, NOTCH and growth factor receptor tyrosine kinase, are associated with the induction of EMT in certain physiological circumstances. TGF-β, a cytokine released by tumor cells and stromal fibroblasts in the TME, is regarded a main cause of EMT (Katsuno et al., 2013). Other signaling pathways involved in EMT induction are inflammatory cytokines such as TNF-a *via* NF-jB (Wu et al., 2009), IL-6/STAT pathways (Lo et al., 2007) and extracellular matrix (ECM) stiffness (Wei et al., 2015). Then these signaling molecules can stimulate various EMT transcribing factors EMT-(tf) to start the EMT program, including inhibition of epithelial markers and stimulation of mesenchymal markers.

The occurrence of CD4^+^ T lymphocyte inflammatory infiltration in UM has been reported. Moreover, the ability of CD4^+^CD25+FoxP3+ Treg cells to suppress Th1 or cytotoxic T lymphocyte reactions is a main principle of tumor escape in many cancers (Amaro et al., 2017). In cardiomyocyte studies, fibroblast growth factor (FGF)-2 generation can be modulated transcriptionally (Jin et al., 2000) and FGF-2 prevents UM cells from growth restriction by bromodomain and extra-terminal protein inhibitors (Chua et al., 2019). In addition, EMT may contribute to the transdifferentiation of epithelial tumor cells, conferring their migration and invasiveness (Smolkova et al., 2018).

In present academic research, two independent UM cohorts were utilized explore the significance of metastasis-associated genes (MAGs) in UM in order to explore new prognostic biomarkers. We set up a MAGs-based risk score system for forecasting prognosis of UM cases. Our data disclosed potential function and prognostic power of MAGs in UM. Furthermore, AREG was selected to confirm the model accuracy by various wet lab experiments.

## Materials and methods

### Data collection

The gene expression profile and relevant clinical data were acquired from the GEO (https://www.ncbi.nlm.nih.gov/geo/) and TCGA (https://portal.gdc.cancer.gov/) databases, respectively. The TCGA-UM cohort including the gene expressions and clinical data of 80 UM patients was chosen as the training set to build a prognostic model. The GSE22138 with RNA sequencing of 63 UM samples was used as the validating set. The metastasis-associated genes (MAGs) obtained from MSigDB website (https://www.gsea-msigdb.org/gsea/index.jsp) are provided in Supplementary Table S1.

### Construction of MAGs-based risk score system (MBRSS)

Prognostic genes in the training set were identified through univariate Cox analysis. Then the coefficients of these model genes were computed to construct a prognostic model *via* multivariate analysis. The equation is: $risk\;score=\sum_{i=1}^{n}\left(coef\times {Exp}_{i}\right)$ , where Exp_i_ and coef are the expression level and risk coefficient of each gene respectively. The patients were classified by the median risk score into high- and low-risk groups. An external dataset GSE68465 was adopted to validate the predictive ability of signature.

### Functional enrichment analysis

GSEA was done to uncover the probable molecular mechanisms of the prognostic genes at the cutoff value of adjusted *p* < 0.05. The signaling pathways for UM were recognized using the Kyoto Encyclopedia of Genes and Genomes (KEGG) on R clusterProfiler and visualized on R ggplot2 (Yu et al., 2012).

### Determination of a prognostic nomogram

The independence of the model was determined *via* Cox regression analyses. Then a nomogram was set up to strengthen the predictive ability of the model based on diverse clinical traits. The nomogram was verified *via* a calibration curves.

### Immune activity analysis

Relative infiltration levels of 21 types of immune cells were quantified using the CIBERSORT algorithm as described before (Subramanian et al., 2005). The immune activities between groups, as de-scribed by the normalized enrichment score (NES), were compared with single sample gene set enrichment analysis (ssGSEA).

### Single-cell analysis

To investigate the expression pattern of genes at single-cell level, GSE139829 dataset including 11 samples was collected from GEO database. We applied “Seurat” R package to conduct data quality control and normalization. The UMAP algorithm was employed to reduce the dimension of data. Next, cells were annotated according to surface markers.

### Cell culture and transfection

Human UM cell line (MUM-2B) was obtained from the Fuheng Biology Inc., (Fuheng, Shanghai, China). For MUM-2B cell culture, DMEM (keyGEN bioTECH, China) with 10% fetal bovine serum (FBS) was used. Cells were transfected with the synthesized siRNAs (GenePharma, China) targeting AREG by the Lipofectamine3000 based on the manufacturer’s protocol. The siRNA-AREG sequences are provided in Supplementary Table S2.

### Quantitative real-time PCR

The cell total RNA of was collected using RNA easy reagent (Vazyme, China) and cDNA was obtained using a PrimeScript RT Reagent Kit (Takara, Japan). Then, qRT-PCR was performed through a ChamQ SYBR qPCR Master Mix (Vazyme, China). The relative expression levels of m RNA were normalized to GAPDH. The primer sequences of AREG and GAPDH are shown in Supplementary Table S1.

#### Cell capability assay

The transfected cells were seeded in a 96-well plate. Cell capability was measured by CellTiter-Glo luminescent cell viability assay (CTG, Promega, Germany). After CTG kit incubation, the luminescence was detected multifunctional enzyme marker.

#### EdU assay

The transfected cells were seeded in a 96-well plate. Following incubation in EdU reagent (Ribobio, China) for 2 h, cells were fixed and permeated, and stained with Apollo reagent for half hour. Nuclei were stained with Hoechst 33342.

#### Migration and invasion assays

A transwell insert with 8 mm pores (Millipore) was utilized. In the upper chamber, 1 × 10^4^ cells were seeded in 200 mL media without serum, while 500 mL complete medium was supplied in the lower chamber. Based on the manufacturer’s instructions, we performed Matrigel for the invasion detection (BD Biosciences, United States).

#### Immunofluorescence (IF) assay

After 30 min of treatment with the blocking solution, cells were incubated with primary antibody (E-cadherin and N-cadherin) overnight. Fluorescence-labeled secondary antibodies and DAPI were then applied for staining.

### Statistical analysis

All statistical analyses were finished on R 4.0.5. The outcomes of UM cases were compared between groups through Kaplan-Meier (KM) analysis. The area under the curve (AUC) generated by ROC analysis was computed to test the modeling accuracy. The AUCs for 1-, 3-, and 5-year survival rates were estimated.

## Results

### MAGs-based molecular subtype in UM

**FIGURE 1:**
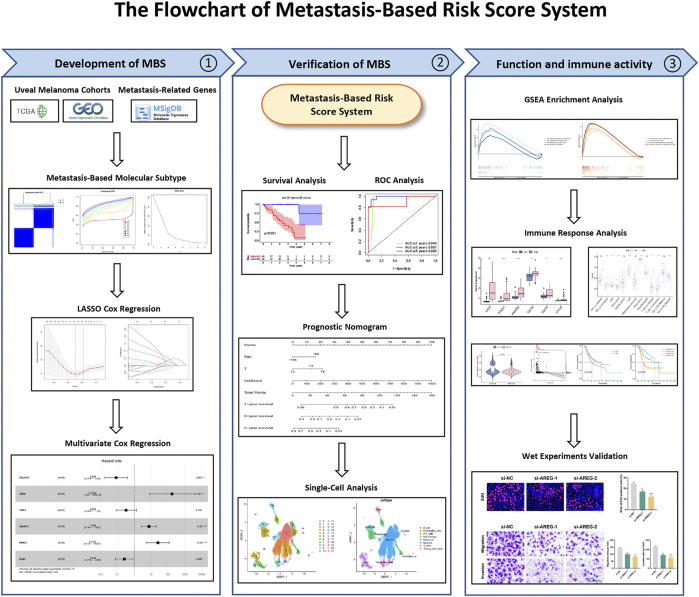
The flowchart of the present research.

The flowchart of the present research is shown in Figure 1.

**FIGURE 2:**
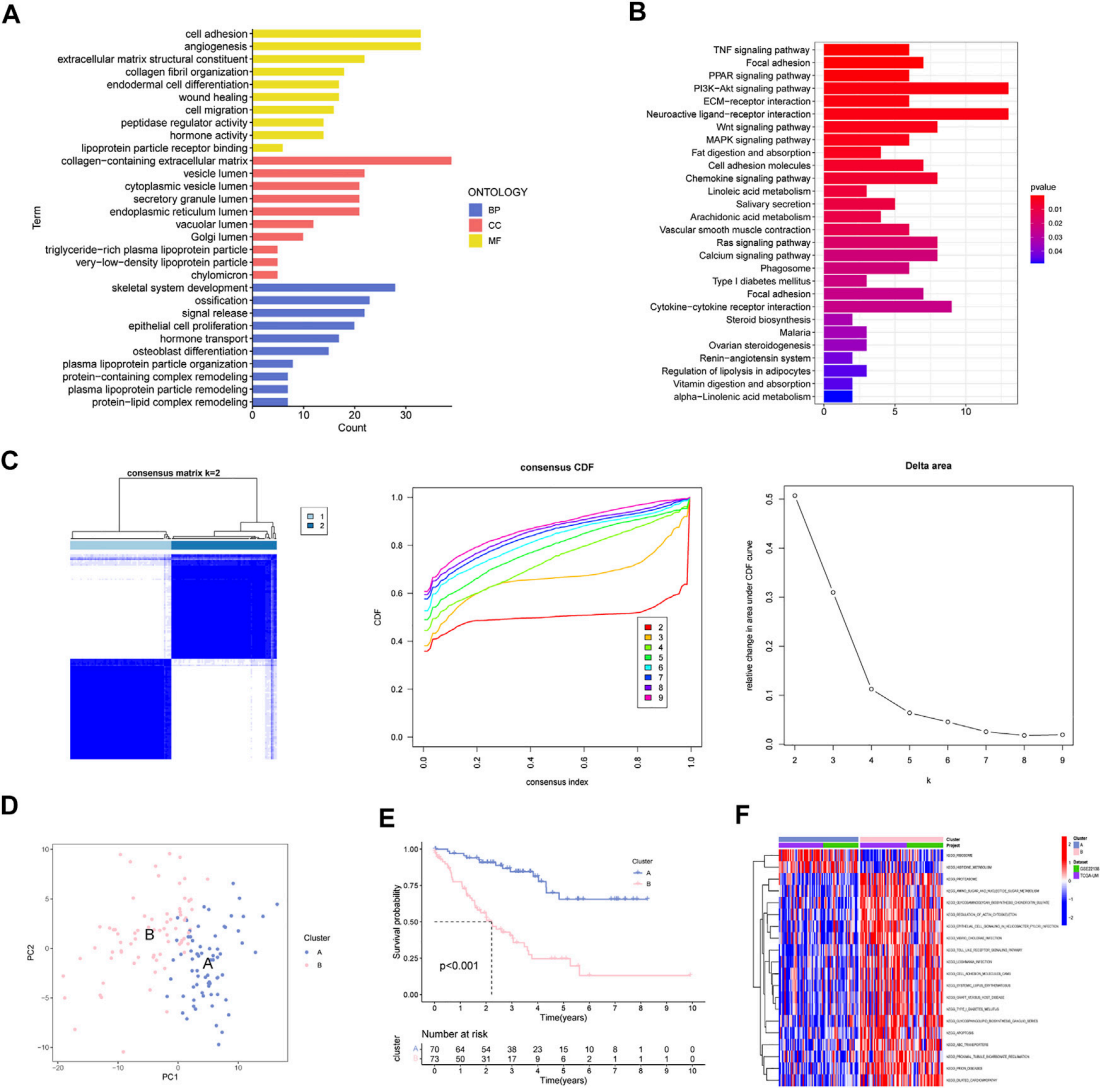
Determination of MAGs-based Molecular Subtype in UM. **(A)** GO analysis, **(B)** KEGG enrichment and **(C)** Consensus clustering analysis of MAGs. **(D)** PCA, **(E)** survival analysis and **(F)** GSVA analysis of two subclusters.

Totally 200 MAGs were collected from MSigDB portal. GO and KEGG analyses were employed to better understand the functions of these MAGs. Results revealed that MAGs were mainly involved in EMT-related biological process, including cell adhesion, wound healing and cell migration (Figure 2A). As shown in Figure 2B, MAGs may regulate TNF, PPAR, and Wnt pathways.

Next, we applied consensus cluster analysis of the 200 MAGs and identified a novel molecular subtype. The UM cases were clustered into two optimal subsets at k = 2 (Figure 2C). PCA demonstrated that the two subsets can be effectively separated by MAGs (Figure 2D). Survival curves suggest that cluster A has a favorable survival outcome compared to cluster B (Figure 2E). In addition, epithelial cell signaling and cell adhesion pathways were great in cluster B (Figure 2F).

### Establishment and validation of the MBRSS

**FIGURE 3:**
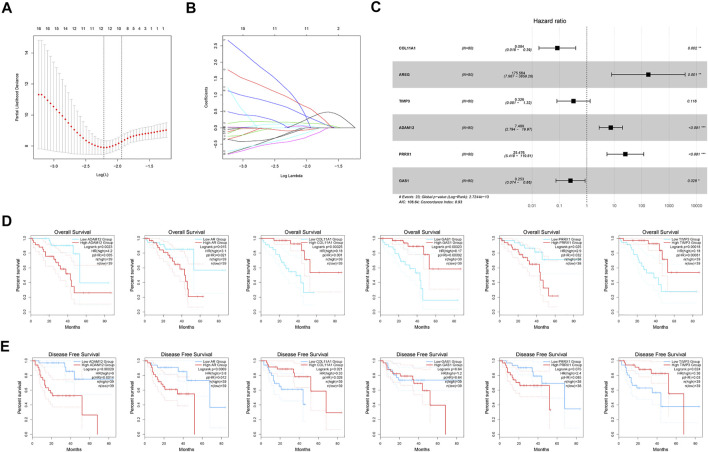
Development of the MAGs-based score system. **(A–B)** LASSO coefficient profile analysis. **(C)** Six MAGs identified for score system (*p < 0.05, **p < 0.01, ***p < 0.001). Kaplan-Meier curves of **(D)** OS and **(E)** DFS.

The training cohort (TCGA-UM) was utilized to screen out prognostic factors. Uni-variate Cox analysis was first applied to determine a total of 94 MAGs with prognostic values. LASSO regression was conducted to shrink the overfitting value of the signature and screened out 12 candidate genes for next analysis (Figures 3A, B). Finally, we obtained six MAGs (COL11A1, AREG, TIMP3, ADAM12, PRRX1, and GAS1) from multivariate Cox analysis to create the MBRSS: [COL11A1 × (−2.4808)] + [AREG × (5.1680)] + [TIMP3 × (−1.1211)] + [ADAM12× (2.0108)] + [PRRX1 × (3.2377)] + [GAS1 × (−1.3746)] (Figure 3C). Depending on the median risk score, all UM samples were divided into high-risk and low-risk groups. KM survival analysis disclosed three protective indicators and three risky indicators (Figure 3D). Except for GAS1 and PRRX1, all other genes were significant in DFS (Figure 3E).

**FIGURE 4:**
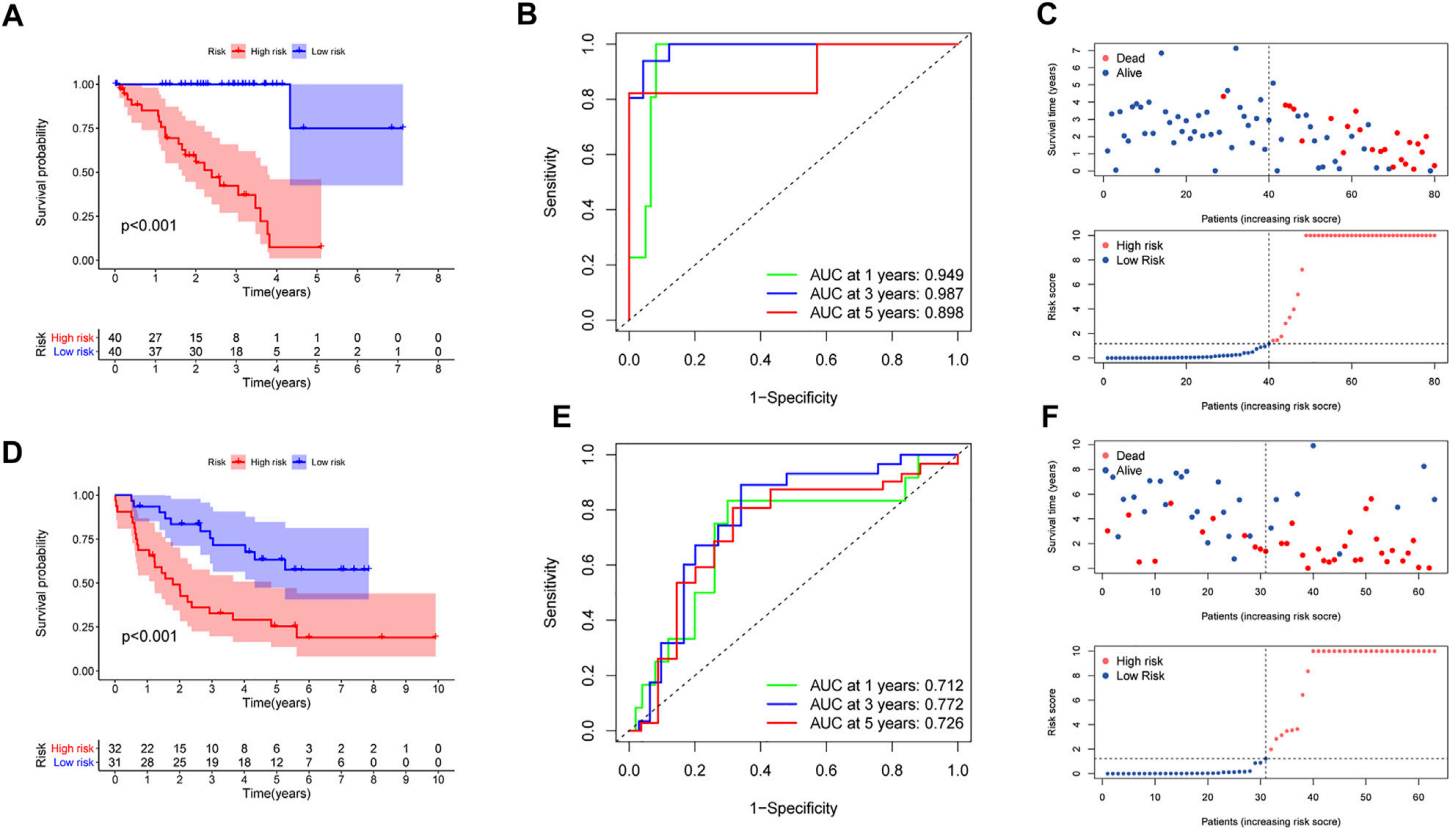
Evaluation of the MAGs-based score system. **(A,D)** Survival analysis for patients in the two subgroups. **(B,E)** ROC curves displayed the favorable ability of the model. **(C,F)** Distribution of the risk score and survival status.

In the training set, K-M curves illustrated that the MBRSS-low subgroup presented favorable survival outcome (Figure 4A). The AUCs of 1-, 3-, and 5-year survival were 0.949, 0.987, and 0.898, respectively (Figure 4B). The risk score and clinical status of each case from two risk groups were shown in Figure 4C. Moreover, we confirmed the forecasting ability of MBRSS in the testing set (Figures 4D–F). The difference in clinical outcome be-tween groups was further verified in the testing cohort. The AUCs were 0.712, 0.772 and 0.726 for 1-, 3-, and 5-year survival, respectively (Figure 4E).

### Development t of an MBRSS-Associated nomogram

**FIGURE 5:**
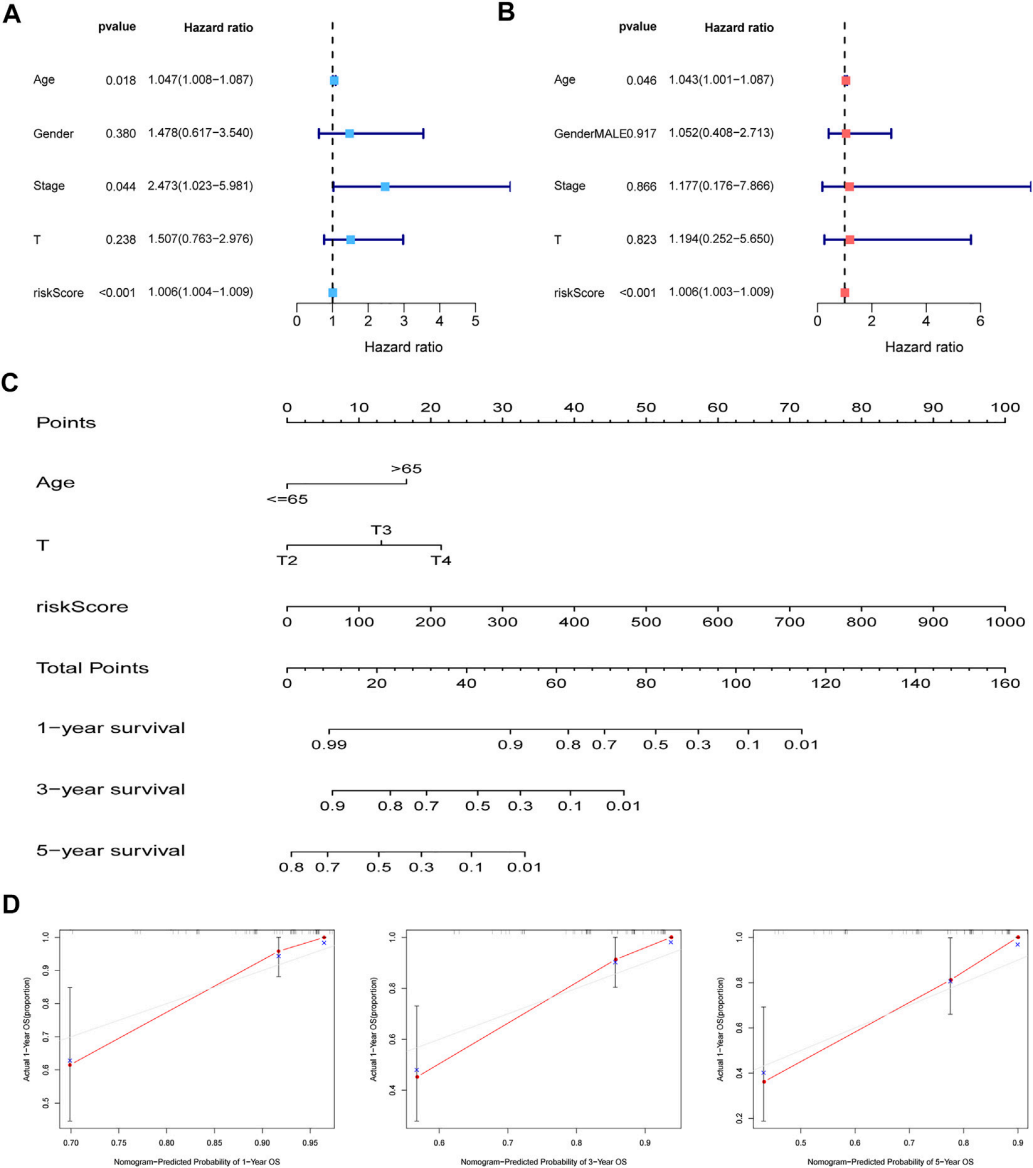
Establishment of the nomogram. **(A–B)** independent prognosis analysis by univariate and multivariate analyses. **(C)** Nomogram for improving prognosis assessment. **(D)** Calibration curves of the nomogram.

Cox regression analysis was performed to confirm the independent value of the MBRSS. Univariate regression unearthed that age, stage and risk score were closely corre-lated to the survival outcome (Figure 5A). After multivariate analysis, the risk score was still an independent prognosis indicator in UM (Figure 5B). Then we set up an MBRSS-based nomogram to enhance its capability of prognosis assessment (Figure 5C). Calibration curves were plotted to demonstrate the optimal forecasting effectiveness of the nomogram (Figure 5D).

### GSEA enrichment of MBRSS

**FIGURE 6:**
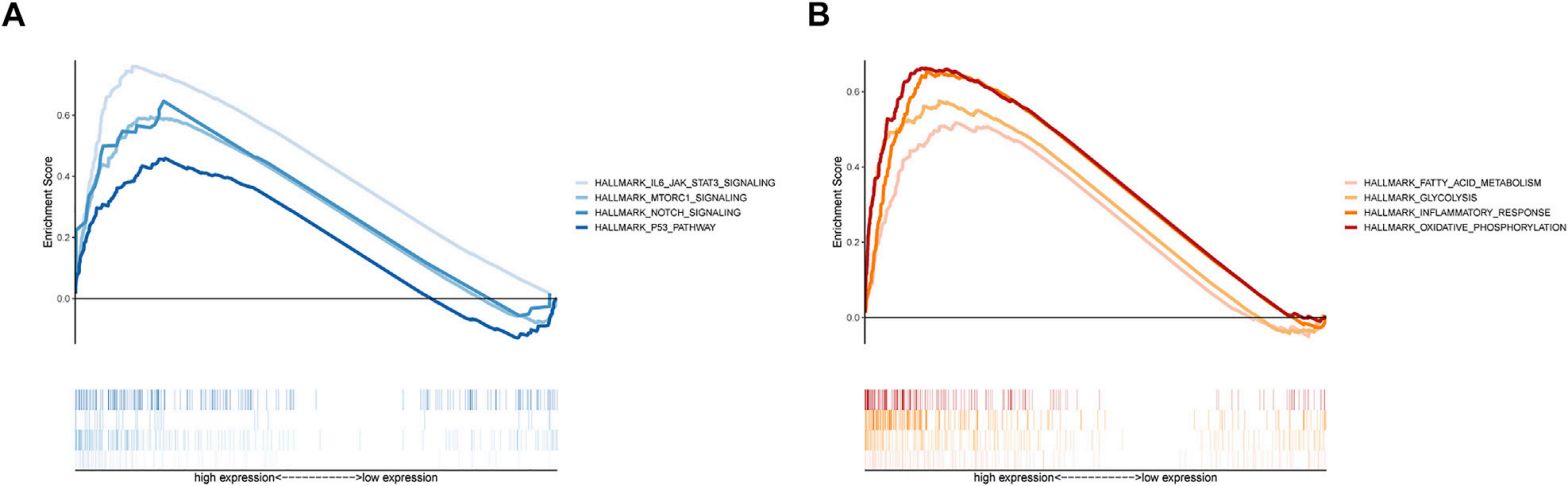
GSEA of MBRSS. **(A)** Tumor-related pathways of hallmark. **(B)** Cellular biological process of hallmark.

GSEA with hallmark gene sets was applied to better understand the underlying functions in the MBRSS-high group. Results disclosed that the high-risk UM samples were related to hallmarks including IL6/JAK/STAT5, mTOR, Notch, and P53 signaling pathways (Figure 6A). In addition, fatty acid metabolism, glycolysis, inflammatory response and oxidative phosphorylation were remarkably enriched in the MBRSS-high group (Figure 6B).

### Characterization of Immune Landscape in UM

**FIGURE 7:**
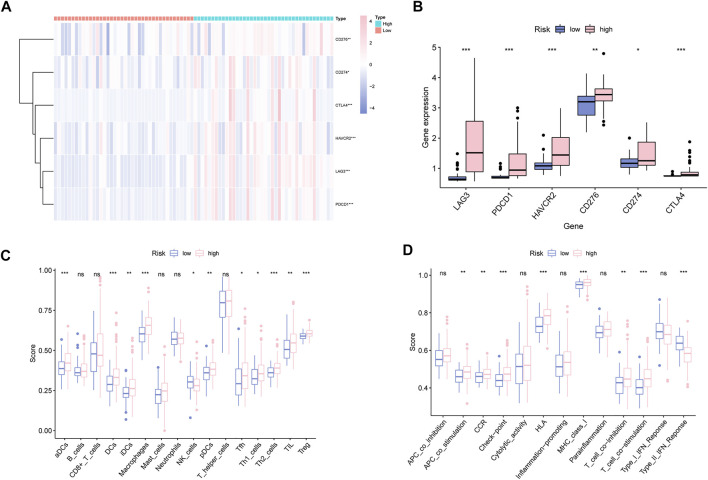
Characterization of Immune Landscape in UM. **(A–B)** Immune checkpoints analysis. **(C)** Immunocyte infiltration analysis. **(D)** The relationship between immune function analysis and MBRSS (ns > 0.05, *p < 0.05, **p < 0.01, ***p < 0.001).

Given the essential effect of immune checkpoints in anti-tumor immunotherapy, their correlation with MBRSS was detected. LAG3, PDCD1, HAVCR2, CD276, CD274 and CTLA4 were highly expressed in the high-MBRSS group (Figures 7A, B). Figure 7C presents the differences in immunocyte infiltration level between the two groups. As for the immune function of UM samples, APC stimulation, checkpoint, HLA, II-IFN response were activated in the high-MBRSS group (Figure 7D).

### Clinical potency analysis of MBRSS

**FIGURE 8:**
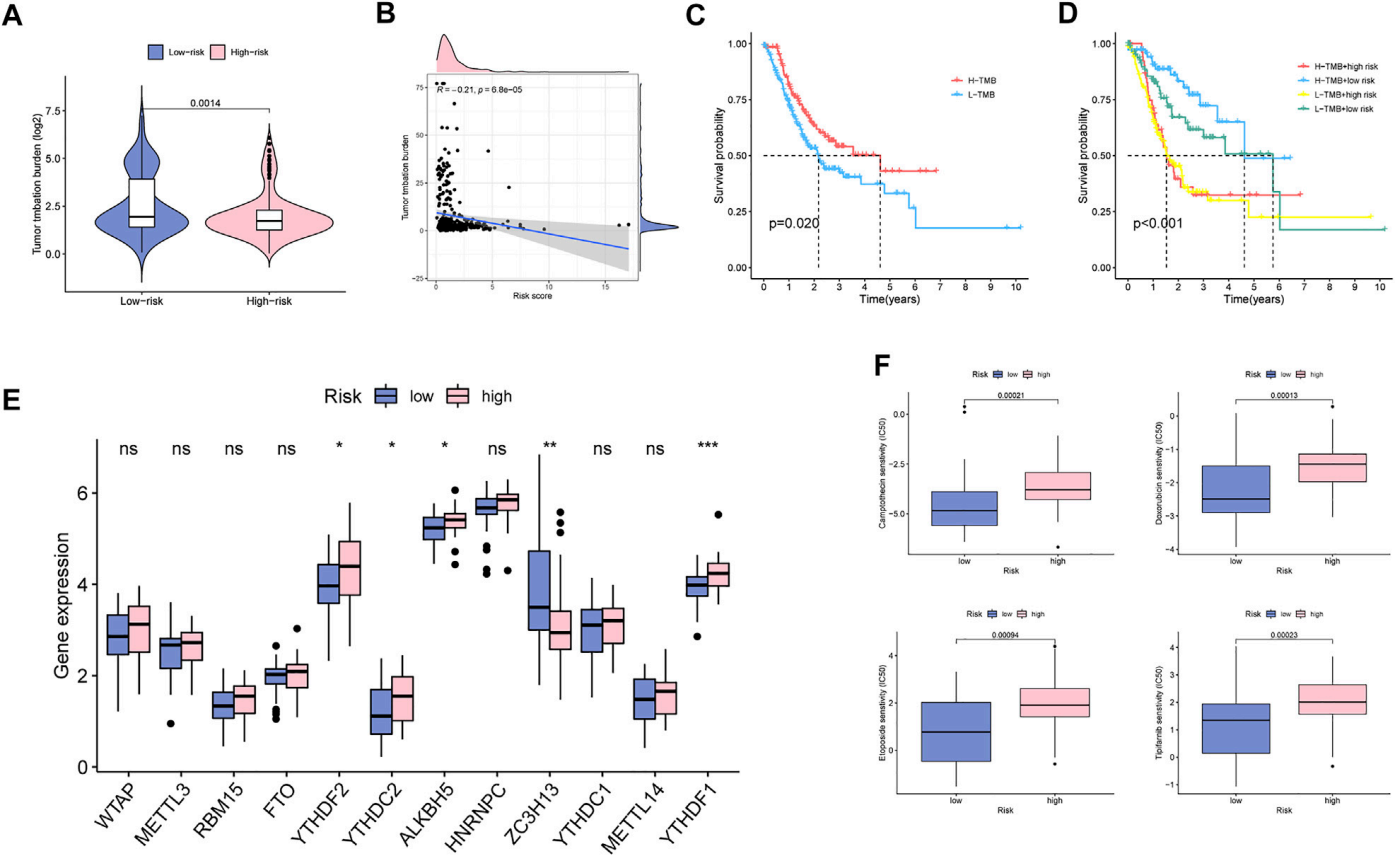
Clinical potency analysis of MBS. **(A–B)** The relationship between TMB and MBRSS. Survival analysis of different groups **(C)** with TMB and **(D)** with TMB and risk score. **(E)** The relationship between m6A regulators and MBRSS (ns > 0.05, *p < 0.05, **p < 0.01, ***p < 0.001). **(F)** Drug sensitivity analysis of MBS.

We further explored the relationship between TMB and MBRSS and found that TMB value was lower in the high-MBRSS group (Figures 8A, B). Survival curves illustrated that the high-TMB UM patients presented favorable survival outcome (Figure 8C). The UM cases with low TMB and high risk had the lowest 5-year survival rate (Figure 8D). In addition, the relationship between MBRSS and m6A regulators was analyzed. Results revealed that YTHDF2, YTHDC2, ALKBH5 and YTHDF1 were upregulated, and ZC3H13 was low expressed in high-MBRSS group (Figure 8E). Drug sensitivity analysis demonstrated that High-MBRSS group displayed high IC50 value of Camptothecin, Doxorubicin, Etoposide and Tipifarnib (Figure 8F).

### Single-cell analysis of MBRSS

**FIGURE 9:**
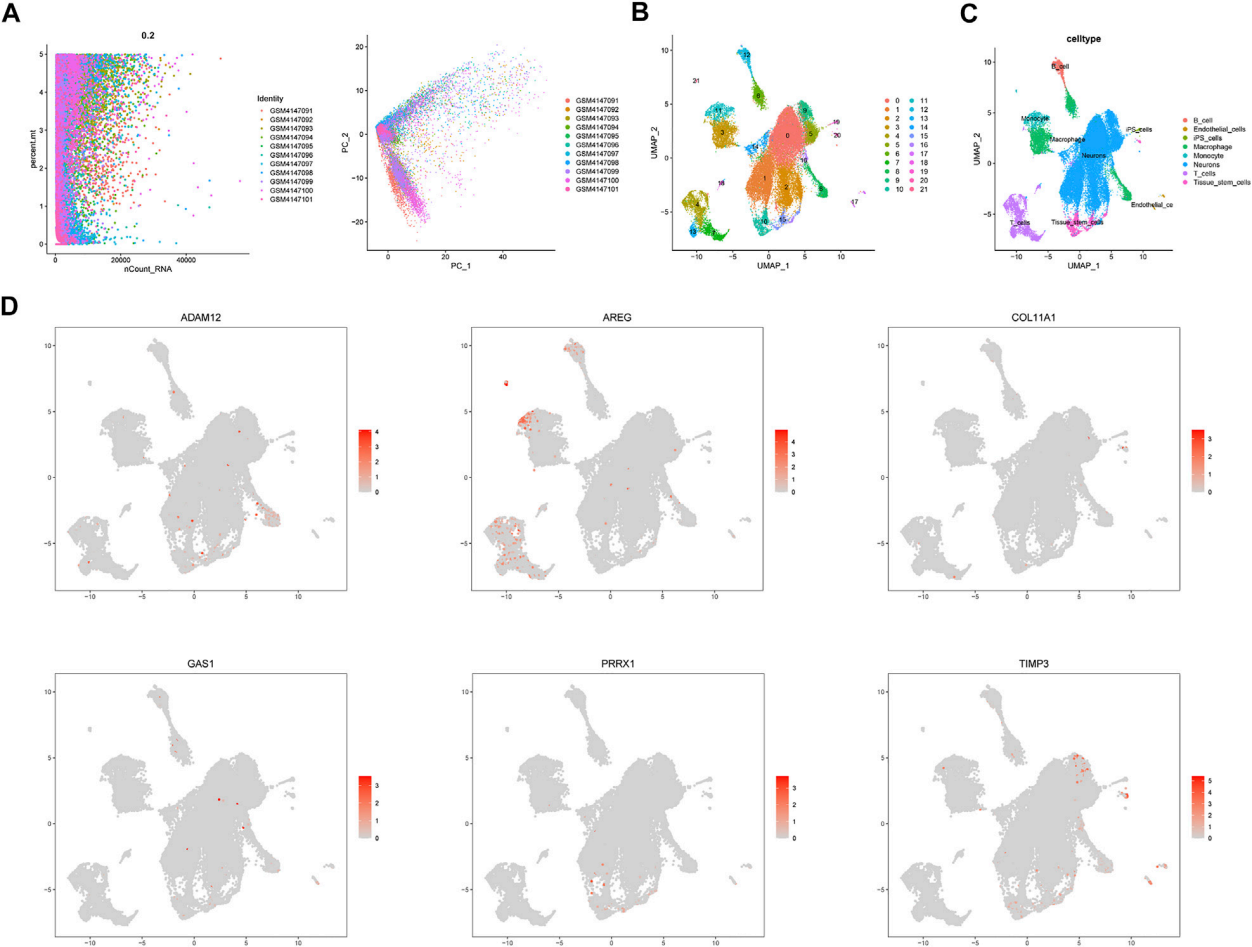
Single cell sequencing analysis. **(A)** Data integration of 11 samples. **(B)** Dimensionality reduction and cluster analysis. **(C)** The cells were classified into approximately 8 cell types, including B cells, endothelial cells, iPS cells, macrophage cells, monocyte cells, neurons, T cells and stem cells. **(D)** Cell location of each model gene.

A total of 11 UM samples were collected from GSE139829. Figure 9A presents a favorable integration effect of 11 samples, suggesting this data can be utilized for next analysis. After dimension reduction and the t-SNE clustering, all cells were divided into 22 different clusters (Figure 9B). According to different cell markers, 22 cell clusters were classified into 8 cell populations including B cells, endothelial cells, iPS cells, macrophage cells, monocyte cells, neurons, T cells and stem cells (Figure 9C). Then, we explored the cell location of each model genes. The results indicated that ADAM12 highly expressed in macrophage cells, AREG mainly located in T cells and TIMP3 highly expressed in neurons (Figure 9D).

### Knockdown of AREG blocks UM proliferation and metastasis

**FIGURE 10:**
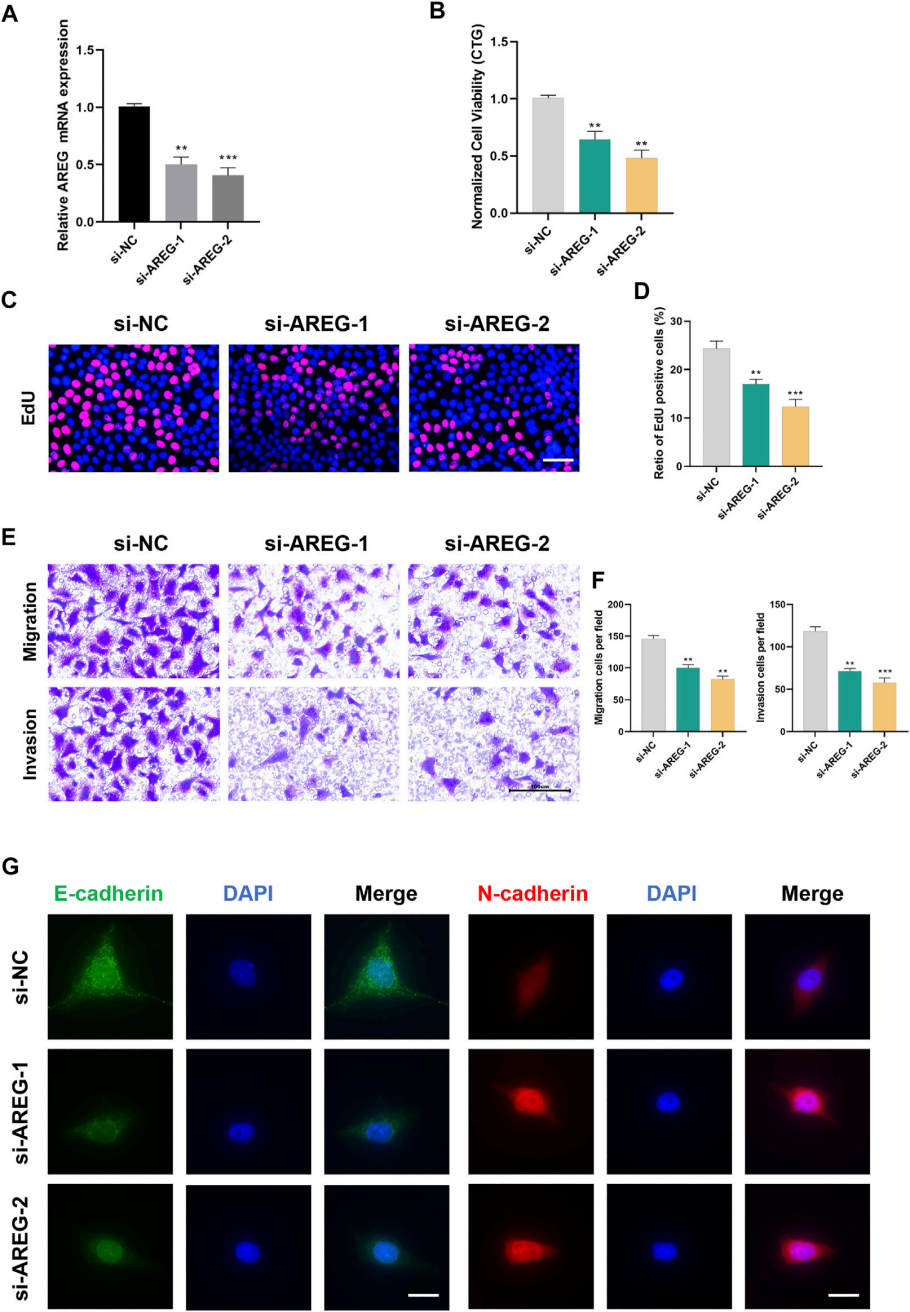
Silencing AREG inhibits UM proliferation and metastasis. **(A)** Transfection efficiency was detected by qRT-PCR. Cell proliferation was detected by **(B)** CTG and **(C,D)** EdU assays. Scale bar, 50 mm. **(E,F)** The role of AREG on cell metastasis was tested by transwell assay. **(G)** Downregulation of AREG enhanced N-cadherin and inhibited E-cadherin by Immunofluorescence. Scale bar, 50 mm (**p < 0.01, ***p < 0.001).

We selected AREG for *in vitro* experiments since it has the highest HR score. Figure 10A shows the favorable silencing efficiency by qRT-PCR assay. Then, we observed that MuM-2B cells proliferation was greatly inhibited by silencing AREG based on the results of CTG and EdU assays (Figures 10B–D). To evaluate the role of AREG on MuM-2B cell metastasis, transwell assay was conducted. The results indicated that cell migration and invasion ability were remarkably suppressed in AREG knockdown group (Figures 10E, F). Then, we explore the role of AREG in regulation of cell metastatic ability by IF assay. The results disclosed that silencing AREG blocked E-cadherin expression whereas enhanced N-cadherin levels, indicating that AREG affects UM cell metastasis through meditation of EMT process (Figure 10G).

## Discussion

We probed into the prognostic characteristics of UM based on comprehensive assay of TCGA and GEO. To investigate the relationship between patient prognosis and gene expression in the training set, we applied KM, univariate Cox analysis, and LASSO Cox regression, which found 10 genetic features associated with prognosis. Applying this signature to the training group, we found significant differences in Cox regression, ROC, and KM analyses between the high- and low-risk groups. The prognostic power of the ten-gene markers was similarly verified in the validation set, which fully demonstrated the validity of the ten-gene signature in forecasting the prognosis of UM. GSEA and immune infiltration analysis suggested that the ten-gene marker risk scores of UM patients may be associated with the TME. This study plays a positive role in guiding the further clinical treatment of UM.

Here, six genes were found to be strongly associated with tumor development. Amphiregulin (AREG) gene, which belongs to the epidermal growth factor (EGF) family, is overexpressed in many cancer tissues. AREG participates in EMT in pancreatic cancer cells through NF-κB signaling and facilitates the movement and spread of pancreatic cancer cells (Wang et al., 2020). AREG upregulates ICAM-1 expression *via* EGFR/PI3K/Akt/NF-κB signaling and promotes the cancer cell viability of osteosarcoma (Liu et al., 2015). Paired related homeobox 1 (PRRX1), is a key member of the homomeric protein pairing family located at the nucleus. PRRX1 mediates cancer cell invasion and metastasis by starting EMT (Meng et al., 2022). In addition, PRRX1 impacts the division and metastasis of various tumor cells *via* Wnt/β-catenin and Notch pathways, and maintains the characteristics of tumor stem cells to promote EMT (Du et al., 2021). A disintegrin and metalloprotease12 (ADAM12) is implied in the starting and advancement of many tumors. ADAM12 is significantly more expressed in hepatocellular carcinoma (HCC) tissues than in surrounding tissues, and a signal pathway related to ADAM12 is found. The high ADAM12 gene expression in HCC tissues is remarkably positively related with T stage, pathological stage and residual tumor (Du et al., 2022). In breast cancer, hypoxia starts HIF-dependent expression of ADAM12, which cleaves the extracellular domain of membrane-bound heparin-bound EGF-like growth factor (HB-EGF). The released extracellular domain of HB-EGF connects to EGF receptor and triggers signal transduction pathways that endow breast cancer cells with enhanced cell migrating and invading abilities, resulting in distant metastasis (Wang et al., 2021). TIMP3 is a main component of the tissue inhibitors of the metalloproteinase (TIMP) family. It is mainly enclosed in the extracellular matrix (ECM) of tissues and inhibits abscission enzymes, transmembrane MMPs and membrane-bound MMPs. TIMP3 promoter methylation is recently recognized as an epigenetic candidate for the treatment of brca1 breast cancer. Knockdown of lncRNA ROR regulates the division, death and invasion of breast cancer cells by inhibiting TIMP3 (Hu et al., 2021). Growth arrest specific 1 (Gas1) plays a key role in growth inhibition. Gas1 negatively regulates glycolysis and provides energy for tumor progression and metastasis. Gas1 negatively regulates the AMPK/mTOR/p70S6K signaling axis and modulates the proliferation, metastasis and abnormal metabolism of malignant tumor cells (Li et al., 2016). Collagen type X1 alpha 1 (COL11A1), from the collagen family, is mostly expressed and released by cancer-related fibroblast subsets, and modulates matrix-tumor interaction and the mechanical characteristics of ECM. It is up-expressed in most human tumor cell lines and tissues and can regulate cell cycle to promote cancer and affect tumor cell proliferation. In ovarian cancer cells, COL11A1 modulates TGF-β3 *via* the NF-κB/IGFBP2 axis, thereby activating cancer-associated fibroblasts and influencing tumor development and migration (Wu et al., 2021).

Immune checkpoints (ICPs), a class of immune-resisting molecules, are expressed on immune cells and mediate the level of immune stimulation. They are pivotal in avoiding autoimmunity (Zhu et al., 2021). ICP molecules enable the immune system to be activated within the normal range. Tumor cells express substances that activate ICPs, which, upon activation, prevent antigen presentation to T cells and in tumor immunity, thereby inhibiting the immune role of T cells and allowing them to avoid surveillance and survive. Immunotherapy through ICPs modulates T cell activity to kill tumor cells through a series of pathways, such as co-inhibition or co-stimulatory signals. UM is a highly metastatic cancer for which ICP therapy is largely ineffective compared to cutaneous melanoma. ICPs are epigenetically mediated *via* DNA methylation. Luka de Vos et al. found that DNA methylation of CTLA4, PD-1, PDL1, PD-L2, LAG3, TIGIT and TIM-3 was remarkably associated with mRNA expressions, BAP1-apoptosis and prognosis of UM. Therefore, the application of ICP gene DNA methylation assays to the biomarker program of the ICP blockade (ICB) trial may help better explain the underlying mechanisms of UM to ICB (de Vos et al., 2022).

The tumor immune microenvironment (TIME) consists of a diverse array of cell types, including T lymphocytes, B lymphocytes, tumor-associated macrophages (TAMs), natural killer cells (NKs), dendritic cells (DCs), tumor-associated neutrophils (TANs), and myeloid-derived suppressor cells (MDSCs). Various biochemical molecules released by the abnormal metabolism of cancer cells reshape the TME and affect the normal immune response of immune cells (Domblides et al., 2019).

Macrophages are important intrinsic immune cells that function mainly through phagocytosis and intake of cellular debris and pathogens, and activation of other immune cells against pathogen invasion. TAMs infiltrating tumor tissues are highly plastic and heterogeneous (Biswas et al., 2013). In the early tumor stage, pro-inflammatory cytokines such as toll-like receptor (TLR) agonists can promote TAM polarization to M-type, and NO and reactive oxygen species (ROS) produced by M macrophages can considerably restrict tumor cell division and kill tumor cells. The tumor cells are killed by NO and ROS (Mantovani and Allavena, 2015). During tumor progression, interleukin (IL)-4 and colony-stimulating factor (CSF)-1 induce TAMs to polarize to M2 macrophages. M2 macrophages secrete EGF, matrix metalloprotein 9 (MMP-9), and other proteins to suppress antitumor effects and promote tumor progression (Mantovani et al., 2017).

Tregs differentiate from initial CD4^+^ T cells, which are a class of immunosuppressive T cells that highly express FOXP3, CD25, and CD4. Tregs often accumulate in tumors, supply energy for immune responses through lipid metabolism and the OXPHOS pathway, maintain the immunosuppression of the TME, and promote tumor infiltration and metastasis (Sharma et al., 2017).

As the first line of antitumor defense in the body, NKs can release perforin on the surface of target cells, resulting in cell perforation, allowing granzyme b to enter tumor cells to induce apoptosis and thus non-specifically kill tumor cells. It also promotes the anti-tumor behaviors of adaptive immune cells by secreting cytokines. Defective transcription factor c-Myc protein (Loftus et al., 2018), accumulation of lactate in the TME (Harmon et al., 2019), and excessive lipid metabolism (Michelet et al., 2018) inhibit the metabolic activity of NKs and affect their normal function.

Type 2 IFN, the main cytokine regulating the immune system, mainly functions to upregulate the expression of MHC molecules and activate macrophages. Type 2 IFN can intensify the activity of NK cells and T cells, promoting the secretion of Thl cytokines, which is conducive to the activation of anti-tumor immune pathway. Additionally, high concentration of type 2 IFN or continuous low dose of type 2 IFN is conducive to the formation of tumor cell immune escape microenvironment. Our results show that type 2 IFN is lowly expressed in the high-risk group and is potentially an early tumor detection and molecular target (Corrales et al., 2017).

Furthermore, we selected AREG to confirm our proposed score system in UM by a variety of *in vitro* experiments. In line with previous studies (Wang et al., 2020; Bolitho et al., 2021), we observed that downregulation of AREG remarkably blocked UM cell growth and metastatic ability, further demonstrating the ability of AREG to regulate proliferation, migration and invasion in tumors.

Nevertheless, there are some shortcomings in our project. Although we performed experiments for validation, the main results were derived from bioinformatics analyses based on public UM datasets. More clinical data from multiple centers need to confirm the ability and accuracy of our proposed MBRSS. Moreover, animal experiments and patient specimens need to further test the role of AREG in UM.

## Conclusion

In conclusion, we successfully identified metastatic molecular subtype in UM and further created a risk score system based on MAGs with single-cell and transcriptome analyses bioinformatics prediction and experimental validation. Further, we found that RRM2 might be a future biomarker and a reference to predict immune response. These findings may aid in understanding the role of RRM2 and its clinical application in cancers.

## Data Availability

The original contributions presented in the study are included in the article/Supplementary Material, further inquiries can be directed to the corresponding authors.

## References

[B1] Amaro A. Gangemi R. Piaggio F. Angelini G. Barisione G. Ferrini S. (2017). The biology of uveal melanoma. Cancer Metastasis Rev. 36, 109–140. 10.1007/s10555-017-9663-3 28229253 PMC5385203

[B2] Arneth B. (2019). Tumor microenvironment. Med. Kaunas. 56, 15. 10.3390/medicina56010015 PMC7023392 31906017

[B3] Augsburger J. J. Corrêa Z. M. Shaikh A. H. (2009). Effectiveness of treatments for metastatic uveal melanoma. Am. J. Ophthalmol. 148, 119–127. 10.1016/j.ajo.2009.01.023 19375060

[B4] Biswas S. K. Allavena P. Mantovani A. (2013). Tumor-associated macrophages: Functional diversity, clinical significance, and open questions. Semin. Immunopathol. 35, 585–600. 10.1007/s00281-013-0367-7 23657835

[B5] Bol K. F. van den Bosch T. Schreibelt G. Mensink H. W. Keunen J. E. Kiliç E. (2016). Adjuvant dendritic cell vaccination in high-risk uveal melanoma. Ophthalmology 123, 2265–2267. 10.1016/j.ophtha.2016.06.027 27476772

[B6] Bolitho C. Moscova M. Baxter R. C. Marsh D. J. (2021). Amphiregulin increases migration and proliferation of epithelial ovarian cancer cells by inducing its own expression via PI3-kinase signaling. Mol. Cell Endocrinol. 533, 111338. 10.1016/j.mce.2021.111338 34062166

[B7] Carvajal R. D. Schwartz G. K. Tezel T. Marr B. Francis J. H. Nathan P. D. (2017). Metastatic disease from uveal melanoma: Treatment options and future prospects. Br. J. Ophthalmol. 101, 38–44. 10.1136/bjophthalmol-2016-309034 27574175 PMC5256122

[B8] Chen F. Song J. Ye Z. Xu B. Cheng H. Zhang S. (2021). Integrated analysis of cell cycle-related and immunity-related biomarker signatures to improve the prognosis prediction of lung adenocarcinoma. Front. Oncol. 11, 666826. 10.3389/fonc.2021.666826 34150632 PMC8212041

[B9] Chua V. Orloff M. Teh J. L. Sugase T. Liao C. Purwin T. J. (2019). Stromal fibroblast growth factor 2 reduces the efficacy of bromodomain inhibitors in uveal melanoma. EMBO Mol. Med. 11, e9081. 10.15252/emmm.201809081 30610113 PMC6365926

[B10] Corrales L. Matson V. Flood B. Spranger S. Gajewski T. F. (2017). Innate immune signaling and regulation in cancer immunotherapy. Cell Res. 27, 96–108. 10.1038/cr.2016.149 27981969 PMC5223230

[B11] Davis F. M. Stewart T. A. Thompson E. W. Monteith G. R. (2014). Targeting EMT in cancer: Opportunities for pharmacological intervention. Trends Pharmacol. Sci. 35, 479–488. 10.1016/j.tips.2014.06.006 25042456

[B12] de Vos L. Carrillo Cano T. M. Zarbl R. Klümper N. Ralser D. J. Franzen A. (2022). , CTLA4, PD-1, PD-L1, PD-L2 , TIM-3, TIGIT, and LAG3 DNA methylation is associated with BAP1 -aberrancy, transcriptional activity, and overall survival in uveal melanoma. J. Immunother. 45, 324–334. 10.1097/CJI.0000000000000429 35862127

[B13] Diener-West M. Reynolds S. M. Agugliaro D. J. Caldwell R. Cumming K. Earle J. D. (2005). Development of metastatic disease after enrollment in the COMS trials for treatment of choroidal melanoma: Collaborative Ocular Melanoma Study Group Report No. 26. Arch. Ophthalmol. 123, 1639–1643. 16344433 10.1001/archopht.123.12.1639

[B14] Domblides C. Lartigue L. Faustin B. (2019). Control of the antitumor immune response by cancer metabolism. Cells 8, 104. 10.3390/cells8020104 30708988 PMC6406288

[B15] Du S. Sun L. Wang Y. Zhu W. Gao J. Pei W. (2022). ADAM12 is an independent predictor of poor prognosis in liver cancer. Sci. Rep. 12, 6634. 10.1038/s41598-022-10608-y 35459884 PMC9033838

[B16] Du W. Liu X. Yang M. Wang W. Sun J. (2021). The regulatory role of PRRX1 in cancer epithelial-mesenchymal transition. Onco Targets Ther. 14, 4223–4229. 10.2147/OTT.S316102 34295164 PMC8291965

[B17] Harmon C. Robinson M. W. Hand F. Almuaili D. Mentor K. Houlihan D. D. (2019). Lactate-mediated acidification of tumor microenvironment induces apoptosis of liver-resident NK cells in colorectal liver metastasis. Cancer Immunol. Res. 7, 335–346. 10.1158/2326-6066.CIR-18-0481 30563827

[B18] Hu A. Hong F. Li D. Jin Y. Kon L. Xu Z. (2021). Long non-coding RNA ROR recruits histone transmethylase MLL1 to up-regulate TIMP3 expression and promote breast cancer progression. J. Transl. Med. 19, 95. 10.1186/s12967-020-02682-5 33653378 PMC7927245

[B19] Jin Y. Sheikh F. Detillieux K. A. Cattini P. A. (2000). Role for early growth response-1 protein in alpha(1)-adrenergic stimulation of fibroblast growth factor-2 promoter activity in cardiac myocytes. Mol. Pharmacol. 57, 984–990. 10779383

[B20] Katsuno Y. Lamouille S. Derynck R. (2013). TGF-β signaling and epithelial-mesenchymal transition in cancer progression. Curr. Opin. Oncol. 25, 76–84. 10.1097/CCO.0b013e32835b6371 23197193

[B21] Li Q. Qin Y. Wei P. Lian P. Li Y. Xu Y. (2016). Gas1 inhibits metastatic and metabolic phenotypes in colorectal carcinoma. Mol. Cancer Res. 14, 830–840. 10.1158/1541-7786.MCR-16-0032 27401611

[B22] Liu J. F. Tsao Y. T. Hou C. H. (2015). Amphiregulin enhances intercellular adhesion molecule-1 expression and promotes tumor metastasis in human osteosarcoma. Oncotarget 6, 40880–40895. 10.18632/oncotarget.5679 26503469 PMC4747375

[B23] Liu J. Geng R. Ni S. Cai L. Yang S. Shao F. (2022). Pyroptosis-related lncRNAs are potential biomarkers for predicting prognoses and immune responses in patients with UCEC. Mol. Ther. Nucleic Acids 27, 1036–1055. 10.1016/j.omtn.2022.01.018 35228898 PMC8844853

[B24] Liu J. Geng R. Zhong Z. Zhang Y. Ni S. Liu W. (2022). N1-Methyladenosine-Related lncRNAs are potential biomarkers for predicting prognosis and immune response in uterine corpus endometrial carcinoma. Oxid. Med. Cell Longev. 2022, 2754836. 10.1155/2022/2754836 35965688 PMC9372539

[B25] Lo H. W. Hsu S. C. Xia W. Cao X. Shih J. Y. Wei Y. (2007). Epidermal growth factor receptor cooperates with signal transducer and activator of transcription 3 to induce epithelial-mesenchymal transition in cancer cells via up-regulation of TWIST gene expression. Cancer Res. 67, 9066–9076. 10.1158/0008-5472.CAN-07-0575 17909010 PMC2570961

[B26] Loftus R. M. Assmann N. Kedia-Mehta N. O'Brien K. L. Garcia A. Gillespie C. (2018). Amino acid-dependent cMyc expression is essential for NK cell metabolic and functional responses in mice. Nat. Commun. 9, 2341. 10.1038/s41467-018-04719-2 29904050 PMC6002377

[B27] Mantovani A. Allavena P. (2015). The interaction of anticancer therapies with tumor-associated macrophages. J. Exp. Med. 212, 435–445. 10.1084/jem.20150295 25753580 PMC4387285

[B28] Mantovani A. Marchesi F. Malesci A. Laghi L. Allavena P. (2017). Tumour-associated macrophages as treatment targets in oncology. Nat. Rev. Clin. Oncol. 14, 399–416. 10.1038/nrclinonc.2016.217 28117416 PMC5480600

[B29] Meng Z. Chen Y. Wu W. Yan B. Zhang L. Chen H. (2022). PRRX1 is a novel prognostic biomarker and facilitates tumor progression through epithelial-mesenchymal transition in uveal melanoma. Front. Immunol. 13, 754645. 10.3389/fimmu.2022.754645 35281030 PMC8914230

[B30] Michelet X. Dyck L. Hogan A. Loftus R. M. Duquette D. Wei K. (2018). Metabolic reprogramming of natural killer cells in obesity limits antitumor responses. Nat. Immunol. 19, 1330–1340. 10.1038/s41590-018-0251-7 30420624

[B31] Mittal V. (2018). Epithelial mesenchymal transition in tumor metastasis. Annu. Rev. Pathol. 13, 395–412. 10.1146/annurev-pathol-020117-043854 29414248

[B32] Sharma P. Hu-Lieskovan S. Wargo J. A. Ribas A. (2017). Primary, adaptive, and acquired resistance to cancer immunotherapy. Cell 168, 707–723. 10.1016/j.cell.2017.01.017 28187290 PMC5391692

[B33] Singh A. D. Turell M. E. Topham A. K. (2011). Uveal melanoma: Trends in incidence, treatment, and survival. Ophthalmology 118, 1881–1885. 10.1016/j.ophtha.2011.01.040 21704381

[B34] Smolkova B. Horvathova Kajabova V. Zmetakova I. Kalinkova L. Czanner G. Markova A. (2018). Role of epigenetic deregulation in hematogenous dissemination of malignant uveal melanoma. Neoplasma 65, 840–854. 10.4149/neo_2018_180420N261 30334454

[B35] Song J. Liu Y. Guan X. Zhang X. Yu W. Li Q. (2021). A novel ferroptosis-related biomarker signature to predict overall survival of esophageal squamous cell carcinoma. Front. Mol. Biosci. 8, 675193. 10.3389/fmolb.2021.675193 34291083 PMC8287967

[B36] Song J. Sun Y. Cao H. Liu Z. Xi L. Dong C. (2021). A novel pyroptosis-related lncRNA signature for prognostic prediction in patients with lung adenocarcinoma. Bioengineered 12, 5932–5949. 10.1080/21655979.2021.1972078 34488540 PMC8806662

[B37] Song J. Zhang S. Sun Y. Gu J. Ye Z. Sun X. (2021). A radioresponse-related lncRNA biomarker signature for risk classification and prognosis prediction in non-small-cell lung cancer. J. Oncol. 2021, 4338838. 10.1155/2021/4338838 34594376 PMC8478572

[B38] Subramanian A. Tamayo P. Mootha V. K. Mukherjee S. Ebert B. L. Gillette M. A. (2005). Gene set enrichment analysis: A knowledge-based approach for interpreting genome-wide expression profiles. Proc. Natl. Acad. Sci. U. S. A. 102, 15545–15550. 10.1073/pnas.0506580102 16199517 PMC1239896

[B39] Wang L. Wang L. Zhang H. Lu J. Zhang Z. Wu H. (2020). AREG mediates the epithelial-mesenchymal transition in pancreatic cancer cells via the EGFR/ERK/NF-κB signalling pathway. Oncol. Rep. 43, 1558–1568. 10.3892/or.2020.7523 32323797 PMC7107775

[B40] Wang R. Godet I. Yang Y. Salman S. Lu H. Lyu Y. (2021). Hypoxia-inducible factor-dependent ADAM12 expression mediates breast cancer invasion and metastasis. Proc. Natl. Acad. Sci. U. S. A. 118, e2020490118. 10.1073/pnas.2020490118 33952697 PMC8126789

[B41] Watnick R. S. (2012). The role of the tumor microenvironment in regulating angiogenesis. Cold Spring Harb. Perspect. Med. 2, a006676. 10.1101/cshperspect.a006676 23209177 PMC3543072

[B42] Wei S. C. Fattet L. Tsai J. H. Guo Y. Pai V. H. Majeski H. E. (2015). Matrix stiffness drives epithelial-mesenchymal transition and tumour metastasis through a TWIST1-G3BP2 mechanotransduction pathway. Nat. Cell Biol. 17, 678–688. 10.1038/ncb3157 25893917 PMC4452027

[B43] Wu Y. Deng J. Rychahou P. G. Qiu S. Evers B. M. Zhou B. P. (2009). Stabilization of snail by NF-kappaB is required for inflammation-induced cell migration and invasion. Cancer Cell 15, 416–428. 10.1016/j.ccr.2009.03.016 19411070 PMC2881229

[B44] Wu Y. H. Huang Y. F. Chang T. H. Chen C. C. Wu P. Y. Huang S. C. (2021). COL11A1 activates cancer-associated fibroblasts by modulating TGF-β3 through the NF-κB/IGFBP2 axis in ovarian cancer cells. Oncogene 40, 4503–4519. 10.1038/s41388-021-01865-8 34117361

[B45] Xue M. Shang J. Chen B. Yang Z. Song Q. Sun X. (2019). Identification of prognostic signatures for predicting the overall survival of uveal melanoma patients. J. Cancer 10, 4921–4931. 10.7150/jca.30618 31598164 PMC6775505

[B46] Yu G. Wang L. G. Han Y. He Q. Y. (2012). clusterProfiler: an R package for comparing biological themes among gene clusters. OMICS 16, 284–287. 10.1089/omi.2011.0118 22455463 PMC3339379

[B47] Zhu Z. Song J. Gu J. Xu B. Sun X. Zhang S. (2021). FMS-related tyrosine kinase 3 ligand promotes radioresistance in esophageal squamous cell carcinoma. Front. Pharmacol. 12, 659735. 10.3389/fphar.2021.659735 34040525 PMC8141745

