# Supplementary material for: Integrated single-cell and transcriptome sequencing analyses develops a metastasis-based risk score system for prognosis and immunotherapy response in uveal melanoma
Source: Front Pharmacol. 2023 Feb 9;14:1138452. doi: 10.3389/fphar.2023.1138452 (PMC9947539; doi:10.3389/fphar.2023.1138452)
Supplement: Supplementary file 2 [file Table2.docx]

Supplemental Table S2

1. The siRNAs targeting AREG were as follows:

si-AREG-1:

5′-GGAUUUGAGGUUACCUCAATT-3′ (sense) and 5′-UUGAGGUAACCUCAAAUCCTT-3′ (antisense);

si-AREG-2:

5′-GCAUGAUUGACAGUAGUUUTT-3′ (sense) and 5′-AAACUACUGUCAAUCAUGCTT-3′ (antisense);

The siRNA control sequences were 5′-UUCUCCGAACGUGUCACGUTT-3′ (sense) and 5′-ACGUGACACGUUCGGAGAATT-3′ (antisense).

2. The primer sequences were as follows:

AREG:

5′-TGAGATGTCTTCAGGGAGTG-3′ (sense) and 5′-AGCCAGGTATTTGTGGTTCG-3′ (antisense);

GAPDH:

5′-GGCATCCTGGGCTACACTG-3′ (sense) and 5′-GTGGTCGTTGAGGGCAAT-3′ (antisense).
